# Genome Sequences of *Frankineae* sp. Strain MT45 and *Jatrophihabitans* sp. Strain GAS493, Two Actinobacteria Isolated from Forest Soil

**DOI:** 10.1128/MRA.00614-20

**Published:** 2020-09-17

**Authors:** Kristen M. DeAngelis, Grace Pold

**Affiliations:** a Department of Microbiology, University of Massachusetts, Amherst, Massachusetts, USA; b School of Natural Science, Hampshire College, Amherst, Massachusetts, USA; c Department of Natural Resources Management and Environmental Sciences, California Polytechnic State University, San Luis Obispo, California, USA; Indiana University, Bloomington

## Abstract

*Frankiaceae* are bacterial endosymbionts that are also found free-living in soil. Here, we present the genome sequences of two novel bacterial members of the order *Frankiales*, class *Actinobacteria*, isolated from temperate terrestrial forest soils. The genomes for MT45 and GAS493 indicate a genetic capacity for carbohydrate degradation but not nitrogen fixation.

## ANNOUNCEMENT

*Frankiaceae* are best known as spore-forming, nitrogen-fixing actinobacteria (1) capable of associating with actinorhizal plants (2), although these plants are not required for their growth (3). Because their role in free-living nitrogen fixation is still unclear, we isolated and sequenced two new taxa that are unclassified in the family *Frankiaceae* and are genomically distinct from known type strains (4).

*Frankineae* sp. strain MT45 and *Jatrophihabitans* sp. strain GAS493 were both isolated from soils collected from an even-aged mixed-hardwood forest stand at the Harvard Forest in central Massachusetts. MT45 was isolated from the organic-rich forest floor, while GAS493 was isolated from the mineral soil below. Isolates were cultivated aerobically on VL55 medium solidified with gellan gum (5) and amended with xylan for MT45 and mixed plant polymers xylan, xanthan, pectin, and carboxymethyl cellulose for GAS493.

Genomic DNA was extracted using the Qiagen Genomic-tip 500/G kit. SMRTbell libraries were constructed and sequenced on the PacBio RS platform at the U.S. Department of Energy (DOE) Joint Genome Institute (JGI) (6), generating 383,007 reads filtered to 122,552 subreads totaling 611.7 Mbp for MT45 and 506,493 reads filtered to 259,823 subreads totaling 914.4 Mbp for GAS493. The libraries had an *N*_50_ value of 4.229 Mb for MT45 and 4.88 Mb for GAS493. The raw reads were assembled using HGAP (v.2.3.0 p5; protocol v.2.3.0, RS HGAP Assembly.3 method, smrtpipe.py v1.87.139483) (7). The final assembly was one 4.229-Mbp scaffold with 70.0× input read coverage for MT45 and one 4.880-Mbp scaffold with 156.0× coverage for GAS493. CheckM (8) indicates that the MT45 and GAS493 genomes are 95.76% and 95.91% complete with 1.4% and 1.5% contamination, respectively. The MT45 and GAC493 genomes have GC contents of 67.3% and 66.94%, respectively.

Genes were identified using Prodigal (9), manually curated using GenePRIMP (10), and then translated to search the NCBI nonredundant database and the UniProt, TIGRFam, Pfam, KEGG, Clusters of Orthologous Groups (COG), and InterPro databases. rRNA genes were found by searches against models of the rRNA genes built from SILVA (11). Gene searches were completed within the JGI Integrated Microbial Genomes (IMG) platform (12) and KBase (13). Default parameters were used for all software unless otherwise noted.

Genomes were compared by average nucleotide identity (ANI) and using Mash (14) in KBase (13), which uses a whole-genome nucleotide clustering method to define genome similarity. GAS493 and MT45 share 84% ANI and a Minhash D of 0.147, or 1 − ANI (14). The next closest relative to GAS493 and MT45 was a *Geodermatophilus* species, which had D values of 0.241 and 0.244, respectively. MT45 has two rRNA operons; the two 16S rRNA genes are identical in sequence and bear 99% identity to those of *Jatrophihabitans* sp. GAS493 and less than 97% to any other cultivated bacteria.

Carbohydrate utilization is observed among only some *Frankiaceae* (1), and both MT45 and GAS493 have dozens of genes annotated for carbohydrate activity (15). Both lack the canonical genes for nitrogen fixation, but they do have a NifU-like gene encoding an Fe-S cluster assembly protein with diverse functions in cell redox (16). Both genomes contain genes required for nitrogen assimilation through ammonium reduction, including an ammonia permease gene, which are observed for free-living diazotrophs (1). Although some *Frankia* spp. can fix nitrogen based on the acetylene reduction assay without NifH homologs detected by PCR amplification (17), the mechanism is unknown. Therefore, we propose a potential role for GAS493 and MT45 as nondiazotrophic polysaccharide degraders in soil.

Together, these genomes suggest multiple mechanisms for growth in soils where nutrients can be poor and sparsely available.

### Data availability.

Raw reads are available for download in the Sequence Read Archive under the accession no. SRX2158410 for *Frankineae* sp. MT45 and accession no. SRX3048269 for *Jatrophihabitans* sp. GAS493. Genome assemblies have been deposited in GenBank under the accession no. LT629697 for *Frankineae* sp. MT45 and accession no. LT907982 for *Jatrophihabitans* sp. GAS493.
